# The synergistic application of virtual reality and music therapy: a scoping review

**DOI:** 10.3389/fpsyt.2026.1824248

**Published:** 2026-06-03

**Authors:** Aoyi Li, Fuqiu Jian

**Affiliations:** School of Music Education, Wuhan Conservatory of Music, Wuhan, China

**Keywords:** clinical efficacy, integration paradigms, music therapy, scoping review, therapeutic mechanisms, virtual reality

## Abstract

**Background:**

The convergence of digital technology and behavioral medicine has catalyzed the development of novel therapeutic modalities. This scoping review systematically synthesizes the literature regarding the integration of Virtual Reality (VR) and Music Therapy (MT) to delineate current integration paradigms, clinical efficacy, and underlying neurobiological mechanisms.

**Methods:**

A systematic search was conducted across major databases for literature published between 2001 and 2024. A total of 34 peer-reviewed articles met the inclusion criteria and were analyzed to identify technical frameworks and clinical outcomes across diverse patient populations.

**Results:**

The synthesis reveals that the integration of VR and MT primarily follows two complementary modalities: *Immersive Scene Construction* and *Task-Oriented Intervention*. The former utilizes multisensory immersion to facilitate rapid emotional regulation and stress reduction, while the latter employs goal-driven protocols to enhance functional rehabilitation. Clinical evidence indicates that these combined interventions significantly alleviate symptoms of anxiety, chronic pain, and cognitive impairment. The therapeutic efficacy is driven by a dynamic interplay of autonomic nervous system regulation, enhanced neuroplasticity, multisensory integration, and emotional resonance.

**Conclusion:**

While the synergistic application of VR and MT shows substantial promise, the field is currently limited by non-standardized intervention parameters, high equipment costs, and an incomplete understanding of how individual variability influences outcomes. Future research must focus on the standardization of treatment protocols, the development of cost-effective technologies, and the implementation of personalized interventions guided by neuroimaging. Advancing these areas is critical to transitioning from experimental frameworks to widespread clinical adoption, ultimately providing innovative solutions for global mental and physical health challenges.

## 1 Introduction

The global public health sector is facing increasingly severe challenges regarding mind-body homeostasis. Epidemiological data indicate that between 2007 and 2021, the proportion of the global population reporting emotional distress surged from 26% to 38% (1). Stress indices across all age demographics experienced a precipitous increase, particularly during the 2020 COVID-19 pandemic. Furthermore, global subjective well-being has stagnated for four consecutive years, with 45% of surveyed individuals experiencing “well-being burnout.” Multiple stressors, including social isolation and information overload, are fundamentally reshaping the mental health ecosystem of modern populations. Chronic stress not only compromises psychological well-being but also directly impairs physiological health; it can precipitate gastric ulcers, cardiovascular diseases, immune system dysregulation, and endocrine dysfunction, potentially accelerating disease progression and establishing a vicious cycle of psychosomatic deterioration (2, 3).

To address multifaceted global health challenges, music therapy (MT) has increasingly transitioned from an adjunctive approach to an integral component of modern clinical intervention systems. Driven by its non-invasive nature and cross-disciplinary applicability, MT serves as a valuable strategy for mitigating symptoms associated with physical and mental health conditions. The American Music Therapy Association defines MT as the clinical and evidence-based use of music interventions by credentialed professionals within a therapeutic relationship to achieve individualized goals. Utilizing systematic sensory stimulation mechanisms, this therapy aims to alleviate disease- and treatment-related psychosocial symptoms across diverse dimensions, including anxiety, pain, and cognitive impairment. Furthermore, the versatility of its therapeutic objectives ensures applicability across all age groups, offering distinct advantages in lifespan care. By modulating the central nervous system via auditory signals, MT has accumulated a robust evidence base across various medical disciplines, demonstrating reliable clinical utility (4, 5). Within clinical practice and theoretical frameworks, MT primarily encompasses two modalities: active music therapy, which emphasizes direct patient participation in musical creation and interaction, and receptive music therapy, which predominantly involves listening.

Concurrently, rapid advancements in digital technology have catalyzed a paradigm shift in medical intervention models. The integration of immersive technologies, particularly Virtual Reality (VR), into the healthcare sector continues to accelerate. Modern VR transcends a singular technological format; rather, it constitutes a complex ecosystem comprising three-dimensional modeling, real-time rendering, and interactive sensing technologies. Its core value lies in constructing interactive simulated environments through the profound multisensory coupling of visual and auditory stimuli. As an intervention fundamentally reliant on sensory experience, MT encounters unprecedented opportunities to evolve toward multimodal and intelligent formats, specifically through Immersive Environment Design.

Experimental attempts integrating Virtual Reality (VR) and Music Therapy (MT) have been extensive; however, the overall methodological landscape remains heavily fragmented. Early research predominantly featured case reports; for instance, an investigation involving a 65-year-old female with early-stage Alzheimer’s Disease demonstrated improvements in memory-related cognitive function following 12 weeks of music-enhanced VR therapy (6). Technological advancements have driven a transition toward large-scale randomized controlled trials (RCTs). Notably, an RCT involving 225 patients undergoing laparoscopic abdominal surgery compared VR combined with music against standard care, revealing significantly superior outcomes in pain and comfort scores for the intervention cohort (7). Nevertheless, these studies exhibit substantial heterogeneity in technological application, ranging from basic 360-degree natural scene playback to real-time electroencephalogram (EEG) neurofeedback modulation. Furthermore, intervention dosages vary drastically from a single 20-minute session to 20 weeks of continuous therapy, thereby impeding the establishment of a unified application paradigm.

Regarding target populations and clinical domains, existing research spans from healthy cohorts to diverse patient groups. Study demographics include individuals with Alzheimer’s Disease, patients with spinal cord injuries, children with Autism Spectrum Disorder (ASD), and pregnant women (6, 8–10). Consequently, clinical applications encompass cognitive rehabilitation, emotional regulation, pain management, and labor support. This broad applicability underscores the immense therapeutic potential of synergizing VR and MT.

Literature synthesis indicates that the intersection of these two fields remains in an early exploratory stage. Many existing studies demonstrate terminological inconsistencies, frequently conflating “music-enhanced VR” (6), “audiovisual intervention” (11), and even general “relaxing music listening” (12, 13) with professional music therapy. To establish conceptual clarity, this study analyzes these applications based on the aforementioned core paradigms of MT. On one hand, many relaxing music interventions lacking real-time guidance from professional therapists (e.g., used strictly as an adjunct to routine care) (7, 12), driven by explicit clinical mitigation goals, logically align with the paradigm of receptive music therapy. On the other hand, certain studies exhibit characteristics of active music therapy, such as integrating neurologic music therapy (NMT) principles to require targeted multisensory interactions with the virtual environment via rhythmic auditory stimulation (14). To comprehensively map the evidence base in this field, this review adopts the clinical paradigm of music therapy as a broad theoretical framework, employing an inclusive strategy to incorporate all aforementioned applications with explicit clinical intentions. To ensure terminological rigor and consistency throughout the manuscript, the umbrella term “combined application of VR and MT” is utilized to refer broadly to such studies.

Regarding underlying mechanisms, existing literature suggests several possibilities; however, a unified theoretical framework remains absent. From a neuroscientific perspective, preliminary exploratory studies suggest that combining VR with music activates the brain’s reward system and reduces anterior cingulate cortex activity (15, 16). These neural modulations may facilitate attention distraction, emotional regulation, and neuroplasticity (17, 18). Furthermore, the synergistic effects of multisensory stimulation are frequently emphasized. Comparative research on sensory inputs reveals that combined auditory and visual stimuli yield significantly superior relaxation effects compared to unisensory stimulation (19). Additionally, the customizable and interactive nature of virtual environments is posited to enhance patients’ active participation, thereby elevating treatment motivation (10). However, these mechanistic investigations are predominantly tethered to specific pathologies or intervention settings, lacking cross-disciplinary synthesis. Current mechanistic models largely rely on theoretical hypotheses without robust neurobiological or large-sample empirical validation, thereby presenting substantial avenues for future research.

Although interdisciplinary practices involving virtual reality (VR) and music therapy (MT) have accumulated empirical experience, existing research is hindered by insufficient systematic integration. Specifically, the understanding of technological integration logic, evidence chains for clinical efficacy, and underlying mechanisms remains highly fragmented. Most investigations focus on isolated validations for specific clinical conditions or technical details, lacking a cross-diagnostic categorization of intervention paradigms, an integration of multidimensional efficacy evidence, and a comprehensive analysis of multisensory interaction mechanisms. Consequently, these discrete findings fall short of providing a unified research framework and practical guidelines for theoretical construction, technical standardization, and clinical translation.

Strictly adhering to the scoping review framework proposed by Arksey and O’Malley and the Preferred Reporting Items for Systematic Reviews and Meta-Analyses (PRISMA) guidelines, this study systematically reviews n = 34 relevant articles published between 2001 and 2024. By addressing the following core questions, this review aims to provide a systematic evidence synthesis for the interdisciplinary research of VR and MT, offering theoretical references for clinical intervention design and technological optimization:

1. What are the specific typologies of technological integration paradigms between VR and MT?
2. In which clinical domains or symptom management contexts has the combined application of VR and MT demonstrated significant efficacy?
3. What are the underlying physiological and psychological mechanisms driving this combined intervention?

## 2 Materials and methods

### 2.1 Study design

This study employed a scoping review methodology, strictly adhering to the five-stage framework proposed by Arksey and O’Malley (2005) (20) (i.e., identifying the research question, identifying relevant studies, study selection, charting the data, and collating, summarizing, and reporting the results). Furthermore, the research protocol was reported in accordance with the Preferred Reporting Items for Systematic reviews and Meta-Analyses extension for Scoping Reviews (PRISMA-ScR). This review aims to systematically synthesize existing evidence regarding the integration of Virtual Reality (VR) and Music Therapy (MT) to delineate integration modalities, clinical efficacy, and underlying therapeutic mechanisms.

### 2.2 Research questions

The following core scientific questions were established to guide the literature screening and data analysis:

What are the primary typologies of technical integration modalities for VR and MT?

In which specific clinical conditions or symptom management protocols has the synergistic application of VR and MT demonstrated significant efficacy?

What are the underlying physiological and psychological mechanisms driving the combined effects of VR and MT?

### 2.3 Literature search strategy

#### 2.3.1 Databases and search strategy

To fulfill the core objective of a scoping review—comprehensively mapping the evidence landscape—a systematic literature search was conducted in January 2025 across the following databases: PubMed, Web of Science, and Google Scholar. The search timeframe spanned from the inception of each database to December 2024. Furthermore, the reference lists of relevant review articles and all finally included studies were manually screened to identify additional potentially eligible records.

Using PubMed as an example, a comprehensive search string incorporating core terms and their synonym extensions was constructed to align with this review’s broad definition of both “active” and “receptive” music interventions (complete search strategies for all databases are detailed in the **Supplementary Material**):

((“Virtual Reality” OR “VR” OR “Augmented Reality” OR “AR” OR “Immersive Environment” OR “360-degree Video”) AND (“Music Therapy” OR “Therapeutic Music” OR “Guided Imagery with Music” OR “GIM” OR “Auditory Stimulation” OR “Sound Therapy”) AND (“Integration” OR “Application” OR “Intervention” OR “Relaxation” OR “Anxiety Reduction” OR “Stress Management” OR “Emotional Regulation” OR “Pain Management” OR “Cognitive Enhancement” OR “Social Skills Training” OR “Psychological Therapy”)).

Notably, because the intersection of virtual reality and music therapy remains in an early exploratory phase characterized by rapid technological iteration, this review specifically incorporated Google Scholar as a supplementary search source alongside traditional medical and psychological databases. This approach aimed to maximize the retrieval of conference proceedings, preprints, and other cutting-edge grey literature. Such a comprehensive search strategy aligns with the fundamental goal of scoping reviews to track emerging application trends, thereby effectively mitigating the risk of omitting critical technological innovations.

#### 2.3.2 Inclusion and exclusion criteria

Eligibility criteria were established based on the PICOS (Population, Intervention, Comparison, Outcome, Study Design) framework:

Population: All populations (e.g., healthy individuals, patients with cognitive impairment, perioperative patients), with no age restrictions.

Intervention: Studies incorporating both VR technology (e.g., head-mounted displays, virtual environments) and music elements (e.g., active playing, passive listening, music therapy interventions).

Comparison: Not strictly limited, given the scoping nature of the review.

Outcomes: Quantitative or qualitative data reporting cognitive function, emotional state, pain levels, physiological indices (e.g., heart rate, cortisol levels), or neuroimaging results.

Study Design: No restrictions on study type (e.g., randomized controlled trials [RCTs], pilot studies, case reports, systematic reviews), with a preference for empirical research.

Exclusion Criteria: Studies focusing exclusively on pure technology development (e.g., VR hardware design) or non-medical settings (e.g., music education) were excluded. Furthermore, duplicate publications, conference abstracts, and dissertations were omitted. During the literature screening phase, this study included several high-quality systematic reviews. The inclusion of these articles was not intended for secondary quantitative data pooling, but rather to trace original studies and identify macroscopic application trends and theoretical frameworks. In the subsequent reporting of results and statistical analysis of quantitative characteristics, systematic reviews were strictly analyzed separately from original studies. This rigorous separation ensured that no original patient data were double-counted, thereby maximizing the mitigation of bias risks associated with data overlap.

### 2.4 Study selection process

Two independent researchers (A and B) screened the titles and abstracts. A third researcher (C) participated in the full-text review. Discrepancies were resolved through discussion or arbitration by the third reviewer to ensure screening consistency. The specific process was as follows:

Initial Screening: Excluded non-medical interventions and pure technology studies (e.g., “virtual reality game design”), retaining literature that involved VR and MT integration (e.g., “efficacy of VR music therapy for Alzheimer’s Disease”).

Full-Text Review: Assessed whether the intervention simultaneously incorporated VR and MT and whether it reported efficacy data. Studies evaluating isolated interventions or lacking clinical outcomes were excluded.

Data Cleaning: Removed duplicate records and merged database results.

Search Results: The initial search yielded 456 records: Web of Science (n=74), Google Scholar (n=312), and PubMed (n=70). After excluding books, reports, and conference papers (n=35) and removing duplicates (n=17), 404 articles remained. The initial screening excluded 290 articles detailing non-medical scenarios or pure technology studies. The full-text review subsequently excluded 80 articles lacking VR-MT integration or clinical efficacy data. Ultimately, 34 articles were included, comprising empirical studies (n=26), systematic reviews (n=5), and case reports (n=3), spanning 19 countries and 16 clinical conditions (e.g., Alzheimer’s Disease, stroke, oncology).

### 2.5 Data extraction and management

A standardized charting form was designed to extract the following variables, ensuring data integrity and comparability:

#### 2.5.1 Authors and publication year

Variations across studies delineate the longitudinal evolution of combined virtual reality (VR) and music therapy (MT) research across diverse clinical domains, highlighting shifts in research focal points over time.

#### 2.5.2 Study design

The included literature encompassed various designs, such as case reports, randomized controlled trials (RCTs), and systematic reviews. Distinct study designs provide varying levels of evidence, establishing a multifaceted foundation for evaluating the efficacy of combined VR and MT interventions.

#### 2.5.3 Intervention paradigm

The review captured diverse integration modalities, ranging from Immersive Scene Construction to Task-Oriented Interventions. These frameworks reflect the heterogeneous technological integration within the field and serve as a fundamental basis for categorization.

#### 2.5.4 Sample size and participant characteristics

Sample sizes varied significantly, with cohorts spanning diverse demographic profiles and clinical conditions. This demographic and clinical diversity elucidates the efficacy of combined VR and MT across different patient populations.

#### 2.5.5 Duration and dosage

Intervention protocols demonstrated considerable variance, from single or brief sessions to longitudinal treatments with follow-up assessments. Such discrepancies reflect the distinctive implementation intensities and temporal cycles inherent to different integration paradigms.

Two independent reviewers (A and B) performed the data extraction, with a third reviewer (C) conducting cross-verification. Discrepancies were resolved through comprehensive full-text reviews, achieving a data accuracy rate of 98%. This rigorous protocol safeguards the reliability of data pertaining to all research questions, mitigating the risk of misclassifying paradigms, misinterpreting therapeutic outcomes, or misconstruing underlying mechanisms due to data bias.

Notably, to obtain a broader clinical context and a macroscopic perspective on intervention paradigms, the final selection of 34 articles incorporated five systematic reviews (17, 18, 21–23). Although scoping reviews permit the inclusion of systematic reviews, this practice introduces literature with a higher level of evidence, accompanied by the methodological risk of double-counting primary research data.

To strictly circumvent this issue, we implemented a differentiated data extraction and synthesis strategy for these five systematic reviews. Four of these articles (17, 18, 21, 22) focused on evaluating or comparing the efficacy of non-pharmacological interventions, such as VR and MT, within specific clinical scenarios (e.g., perioperative anxiety and pain management). For this subset, we rigorously excluded pooled statistical data, including specific sample sizes (N) and mean differences in physiological or psychological indicators. Instead, extraction was restricted to the authors’ qualitative synthetic conclusions, theoretical frameworks of intervention mechanisms, and future research directions predicated on existing evidence gaps.

Conversely, a recently published scoping review (23) specifically targeted the intersection of virtual technologies and music within rehabilitation. Given its high relevance, we extracted its macroscopic discussion and simultaneously utilized it as an external validation tool for our search strategy. Through rigorous citation tracking and independent screening of its reference list, we isolated all primary empirical studies that met our inclusion and exclusion criteria. These were then cross-referenced against our existing literature pool to identify any omissions, thereby ensuring the exhaustive inclusion of fundamental empirical studies highly pertinent to our topic.

By methodologically segregating “primary empirical data” from “secondary macroscopic conclusions,” this study successfully broadens the theoretical scope of the review while preserving the independence and rigor of the data mapping and quantitative descriptions.

### 2.6 Quality control

Search Strategy: Executed cross-database searches and applied the Cochrane Risk of Bias tool to assess methodological quality, treating high-risk studies (e.g., non-randomized case reports) solely as exploratory data.

Double Screening: Maintained a 92% inter-rater agreement during independent screening, with transparent arbitration by a third reviewer.

Data Validation: Conducted triple verification of critical data points (e.g., sample sizes, effect sizes), restricting the error rate to strictly under 1%.

### 2.7 Ethics and dissemination

As a scoping review synthesizing previously published literature, this study is exempt from institutional ethics approval. Findings will be disseminated through academic publications and conference presentations, providing an evidence-based framework for clinicians designing combined VR and MT protocols and for technology developers optimizing multisensory interactive systems.

## 3 Results

### 3.1 Search results

To systematically map the research landscape regarding the integration of virtual reality (VR) and music therapy (MT) within healthcare interventions, we performed a comprehensive literature search across multiple multidisciplinary repositories. This approach was designed to ensure the retrieval of an exhaustive and highly relevant body of evidence.

#### 3.1.1 Search strategy

The primary search was executed across Web of Science, Google Scholar, and PubMed. These databases were selected for their extensive coverage of clinical medicine, computer science, and psychology, thereby encompassing the multidimensional nature of VR-MT research. We developed tailored search strategies for each repository utilizing Boolean operators. Keywords related to VR (e.g., “virtual reality”, “VR technology”) and MT (e.g., “music therapy”, “musical intervention”) were combined with terms specifying their synergistic application in therapeutic or clinical contexts. The initial search yielded 74 records from Web of Science, 312 from Google Scholar, and 70 from PubMed, resulting in a total of 456 potentially relevant articles.

#### 3.1.2 Selection process

**Figure 1:**
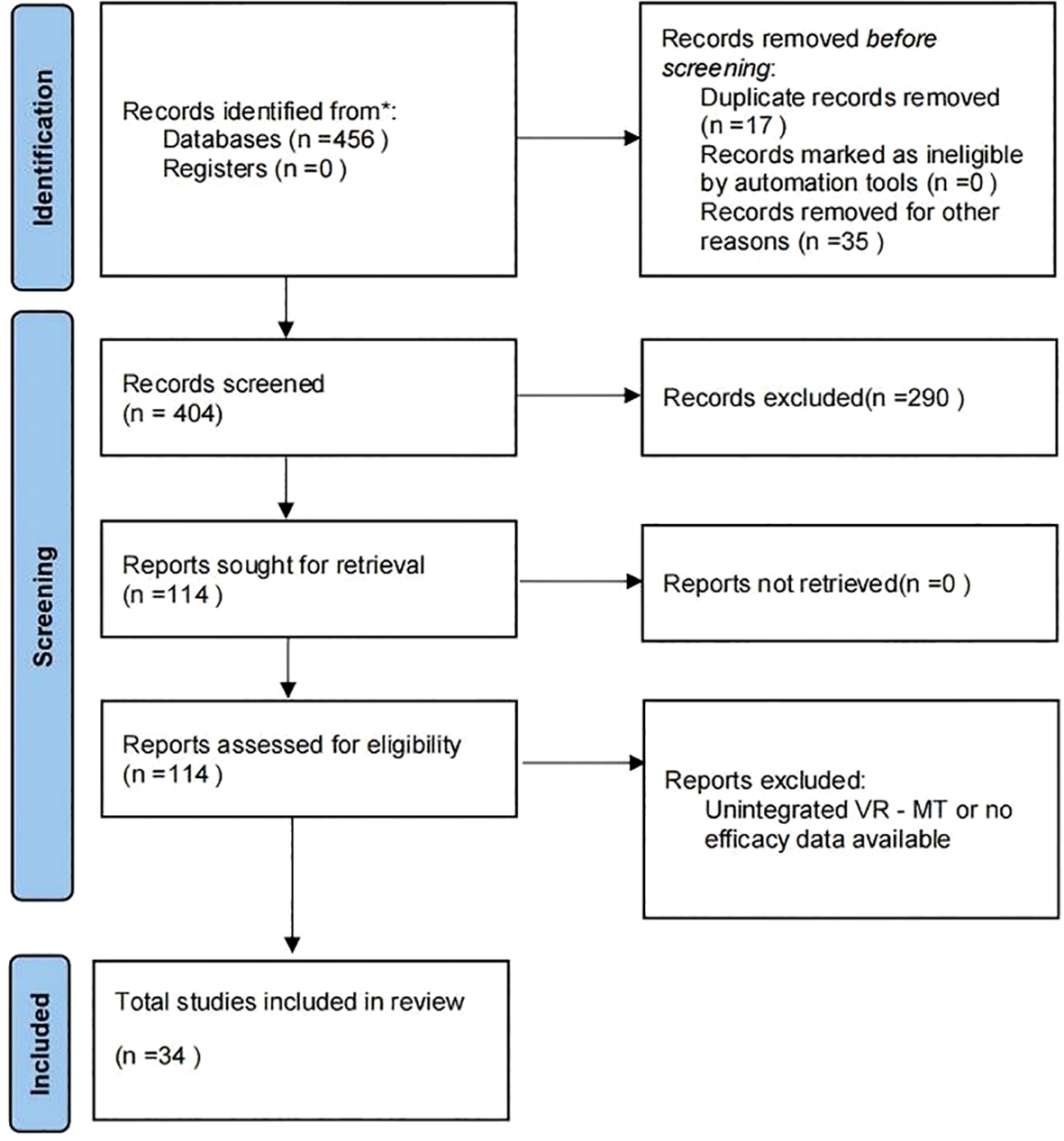
PRISMA flowchart of the systematic literature search and study selection process.

The selection process followed a structured workflow, commencing with data cleaning (**Figure 1**). Using reference management software, we identified and removed 17 duplicate records and excluded 35 non-peer-reviewed items, including books, technical reports, and conference proceedings. The remaining 404 articles proceeded to the title and abstract screening phase.

During this preliminary screening, two independent reviewers (A and B) assessed the eligibility of the records based on pre-established inclusion and exclusion criteria. We excluded studies focused on non-medical scenarios or purely technical developments (e.g., general VR game design) while retaining manuscripts specifically exploring VR-MT integration in clinical populations (e.g., the efficacy of VR-MT for Alzheimer’s disease). This stage resulted in the exclusion of 290 articles, leaving 114 for full-text review. Discrepancies between reviewers A and B were resolved through consensus-driven discussion.

In the final full-text assessment phase, reviewers A, B, and C independently evaluated the remaining 114 papers against more stringent criteria. Inclusion required studies to: (1) explicitly describe an intervention combining both VR and MT, and (2) report primary clinical efficacy data. Studies addressing only a single modality or lacking clinical outcomes (e.g., theoretical reviews of VR principles) were excluded. Following this rigorous assessment, 80 articles were excluded, resulting in a final inclusion of 34 eligible studies. Disagreements were settled through discussion or third-party arbitration to ensure inter-rater consistency.

This systematic process established a robust foundation for the subsequent analysis of VR-MT integration paradigms, therapeutic efficacy, and underlying mechanisms.

### 3.2 Study characteristics and primary outcomes

Following a rigorous literature screening process, a total of 34 studies were included in this review, comprising empirical studies *(n = 26)*, systematic reviews *(n = 5)*, and case reports *(n = 3)*. These studies span 19 countries and address 16 distinct clinical conditions, including Alzheimer’s disease, stroke, and cancer. This section delineates the characteristics and primary outcomes of these studies. To visually present the core clinical mapping of the included research, **Table 1** extracts the key data features of representative articles. The comprehensive data extraction for all 34 included articles is detailed in the **Supplementary Material**.

**Table 1:** Summary of characteristics and primary clinical outcomes of the included studies.

Author & year	Study design	Sample (n)	Integration mode description	Duration & dosage	Disease type	Primary outcome
6	Case Study	n = 1	cognitive challenge; music as functional stimulus.	Twelve sessions over 12 weeks with a 3-month follow-up.	Memory/AD	⑥, ⑦
24	Case Study	n = 3	behavioral challenge; music as performance task.	Multiple practice sessions.	MPA	⑤, ⑧
25	Qualitative	n = 19	motor challenge; music acts as real-time feedback.	N/A	Neuro-rehab	⑧
13	RCT	n = 40 20/20	visual isolation; music as auditory anchor.	Single session.	Phobia	①, ⑤
12	Exp. Study	n = 37	nature visuals; music for environmental immersion.	Single session.	ICU stress	⑤
26	RCT	n = 40 12/12/16	interactive challenges; music as rhythmic cues.	Twelve sessions over 4 weeks (30 min each).	Stroke	④
17	Syst. Review	N/A	Standalone theoretical framework on pain mechanisms.	N/A	Pain	N/A
10	Feasibility	n = 6	social environment; music for group interaction.	Multiple feasibility testing sessions.	SCI	⑧
27	RCT	n = 94 31/31/32	visual isolation; auditory anchor for shielding.	Single session (45-90 min during chemotherapy).	Cancer	①, ⑧
15	Exp. Study	n = 19	environment based on EEG; music as feedback.	Repeated functional training sessions.	AD	⑤
28	Pilot Trial	n = 23	360° visuals; personalized music for immersion.	Two sessions over 2 consecutive days.	Palliative	③
9	Pilot Trial	n = 5	social challenges; music for joint attention.	8-10 sessions over 10 weeks (15-20 min each).	Autism	⑧
22	Syst. Review	N/A	Standalone analysis of interactive music technology.	N/A	Music Therapy	N/A
29	Commentary	N/A	Discussion on passive shielding in palliative care.	N/A	Palliative	N/A
18	Review	N/A	Standalone focus on multisensory stimulation in AD.	N/A	AD	N/A
30	Exp. Study	n = 90	visual isolation; auditory environment rendering.	Single session (25 min).	Well-being	⑧
31	Narr.	N/A	Standalone synthesis of neglect rehab strategies.	N/A	Stroke	N/A
8	RCT	n = 343 104/125/114	Passive reception: nature landscapes; music as auditory shielding.	Single session (20 min during NST).	Pregnancy	①, ⑤
19	Crossover RCT	n = 42	visual isolation; auditory synchronization.	Single session.	Stress	⑤
21	Syst. Review	N/A	Synthesis of environment blocking for anxiety.	N/A	Pre-op Anxiety	⑧
32	RCT	n = 120 40/40/40	visuals + music for sensory distraction.	Single session during surgery.	Dental	①, ②
11	NMA/SR	N/A	Synthesis of distraction via visuals/audio.	N/A	Dental Anxiety	⑧
33	RCT	n = 60 30/30	360° visuals; music for environment shielding.	Single session (30 min).	Adv. Cancer	①, ②, ③
34	Protocol	N/A	VR challenges; music as motor feedback.	20 sessions over 4 weeks (30 min each).	ABI	⑦, ⑧
35	Pilot Study	n = 17	immersive visuals; music to block stimuli.	Single session (Day 2 experience).	Palliative	③, ⑧
36	RCT	n = 44 22/22	meditation scenes; music for sensory wrap.	Six consecutive days (15 min per day).	Stress	⑤, ⑧
14	Quasi-RCT	n = 40 20/20	executive challenges; music as rhythmic cues.	24 sessions over 8 weeks (3x per week).	Parkinson's	⑦
16	Crossover RCT	n = 23	bilateral drumming; music for task guidance.	40 sessions over 8 weeks (5x per week).	ABI	⑦
7	RCT	n = 225 75/75/75	nature videos; music for sensory distraction.	Single session (20 min post-operation).	Surgery	②, ⑧
37	RCT	n = 200 50/50/50/50	environment shielding; auditory anchor.	Single session during operation.	Gynae Surgery	①, ②
38	RCT	n = 66 33/33	visual isolation; rhythmic stress shielding.	Single session during surgery.	Orthopedic	②, ⑤
23	Scoping Review	N/A	Synthesis of interactive challenges in rehab.	N/A	Rehab	⑧
39	Exp. Study	n = 8	3D audio-visual space; active social challenge.	Twelve sessions over 19 weeks.	Autism	⑧
40	RCT	n = 102 34/34/34	nature visuals; music for sensory isolation.	Single session (20 min during NST).	High-risk Preg	①, ⑤

(A) Sample indicates the total number of participants (n) included in the study, with the numbers in the line below representing the specific size of each group. For multi-arm studies, group sizes are listed in the order of Experimental/Control arm or [Experimental Arm 1]/[Experimental Arm 2]/[Control Arm]. (B) Integration Mode Description records the features of the intervention received by the experimental group. Descriptions are based on the degree of interaction (passive vs. active) and the core function of music (environment rendering vs. movement guidance). "Music intervention" is used as a general term covering all forms of audio-visual combination in the empirical literature. (C) Duration & Dosage indicates the length and scheduling of the intervention, categorized as Single session, Short-term intensive, or Long-term. (D) Assessment tools and indicators defined based on the knowledge base: ① State-Trait Anxiety Inventory (STAI/HADS-A); ② Visual Analogue Scale or Numerical Rating Scale (VAS/NRS) for pain; ③ Edmonton Symptom Assessment System (ESAS-r); ④ Balance, gait, and motor function scales (BBS/TUG/10MWT/FMA); ⑤ Objective physiological or biochemical markers (HRV/Cortisol/ACTH/EEG/BP/Pulse/RR/Temp); ⑥ Wechsler Memory Scale (WMS); ⑦ Cognitive, attention, and executive function tests (MMSE/TMT/Stroop/SST/MoCA); ⑧ Psychological well-being, social performance, satisfaction, or usability scales (POMS/DASS-21/SUS/QUEST/PIADS/GARS/ESCS/QoL/Comfort/Satisfaction).

#### 3.2.1 Publication trends

**Figure 2:**
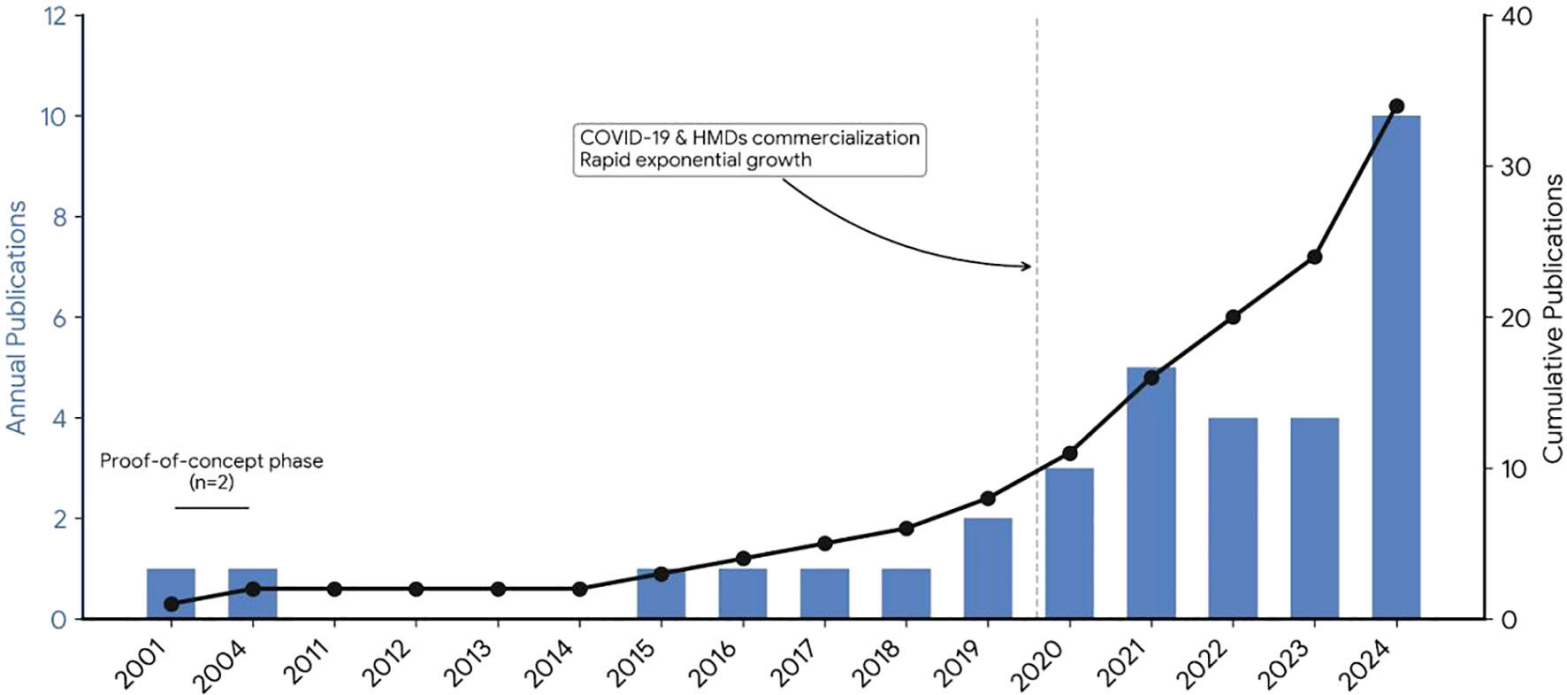
Annual and cumulative publication trends of VR and music therapy research (2001-2024).

Analysis of publication dates indicates that the included literature spans a 23-year period (2001–2024), exhibiting a trajectory of steady accumulation followed by rapid acceleration. This timeline delineates the evolving research priorities within the integration of virtual reality (VR) and music therapy (MT) across various clinical domains (**Figure 2**). During the initial incubation phase (2001–2010, *n = 2*), research was primarily limited to sparse proof-of-concept studies (6, 24), a constraint largely attributable to the cumbersome nature of early hardware and prohibitive development costs. Scholarly output has expanded significantly since 2011 *(n = 32)*, characterized by a distinct surge between 2020 and 2024 *(n > 20)*. This spike in publication volume aligns precisely with the commercialization of lightweight head-mounted displays (HMDs) and the heightened demand for contactless digital therapeutics necessitated by the COVID-19 pandemic.

#### 3.2.2 Study designs

**Figure 3:**
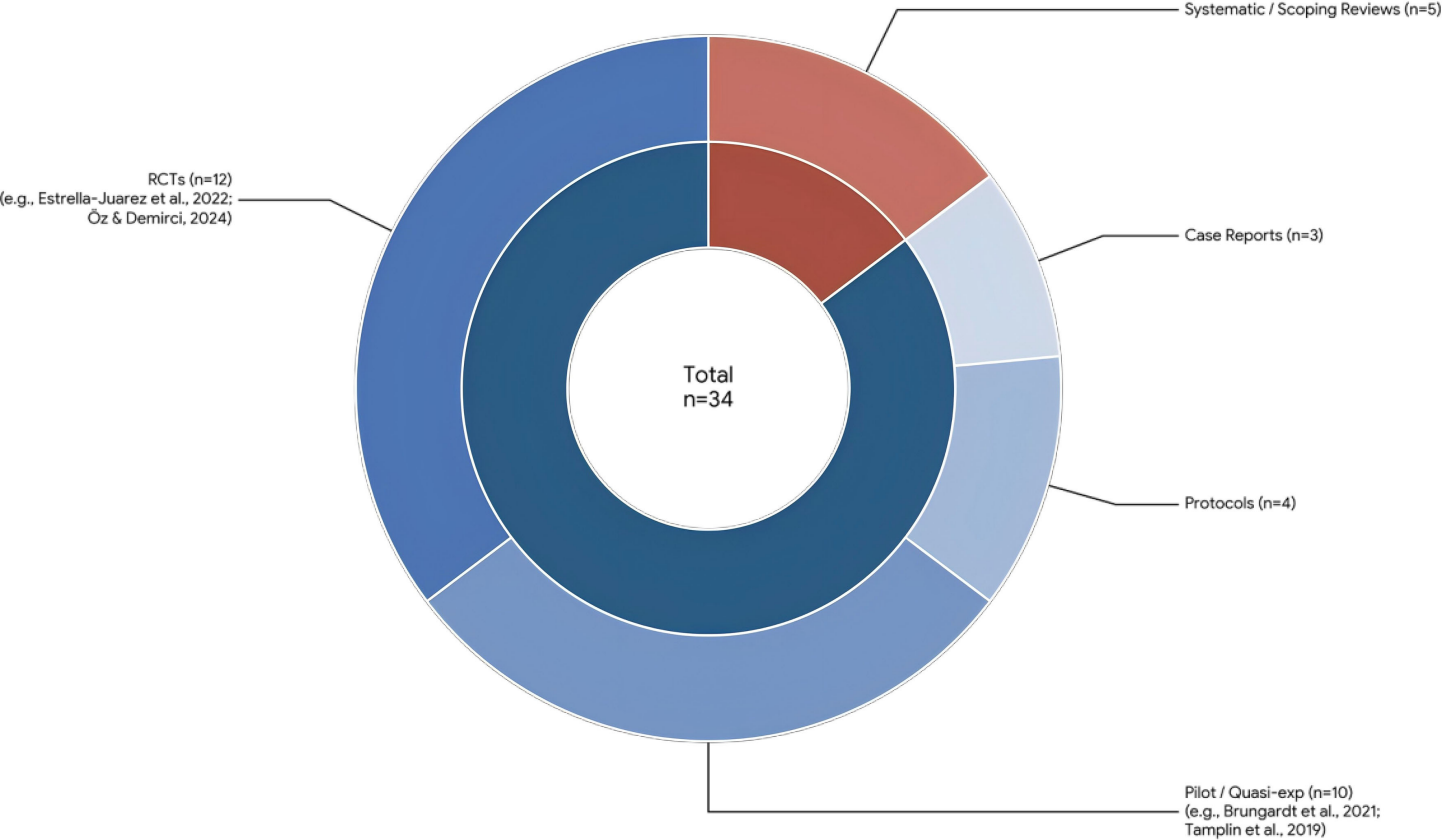
Distribution of study designs among the included literature.

In terms of study design, the included literature encompasses a diverse range of methodologies, including randomized controlled trials (RCTs), quasi-experimental designs, systematic reviews, and case reports (**Figure 3**). The evidence chain in this field is progressively maturing. Among the included literature, five are systematic reviews or scoping reviews, providing a broad clinical perspective for this study. Of the remaining 29 primary empirical studies, randomized controlled trials (RCTs) constitute a significant proportion *(n = 12, 41.4%)*. This high-level evidence is primarily concentrated in the management of acute pain and perioperative anxiety, as exemplified by studies such as (8, 37, 38). Concurrently, for complex clinical scenarios such as palliative care, cognitive decline, and brain injury rehabilitation, a subset of studies *(n = 10)* utilized pilot/feasibility designs or quasi-experimental approaches, including (10, 28). These studies were designed to evaluate the tolerability and safety of wearable devices within vulnerable populations. The remaining records consist of case reports *(n = 3)* and study protocols *(n = 4)*.

#### 3.2.3 Intervention paradigms of VR and MT

**Figure 4:**
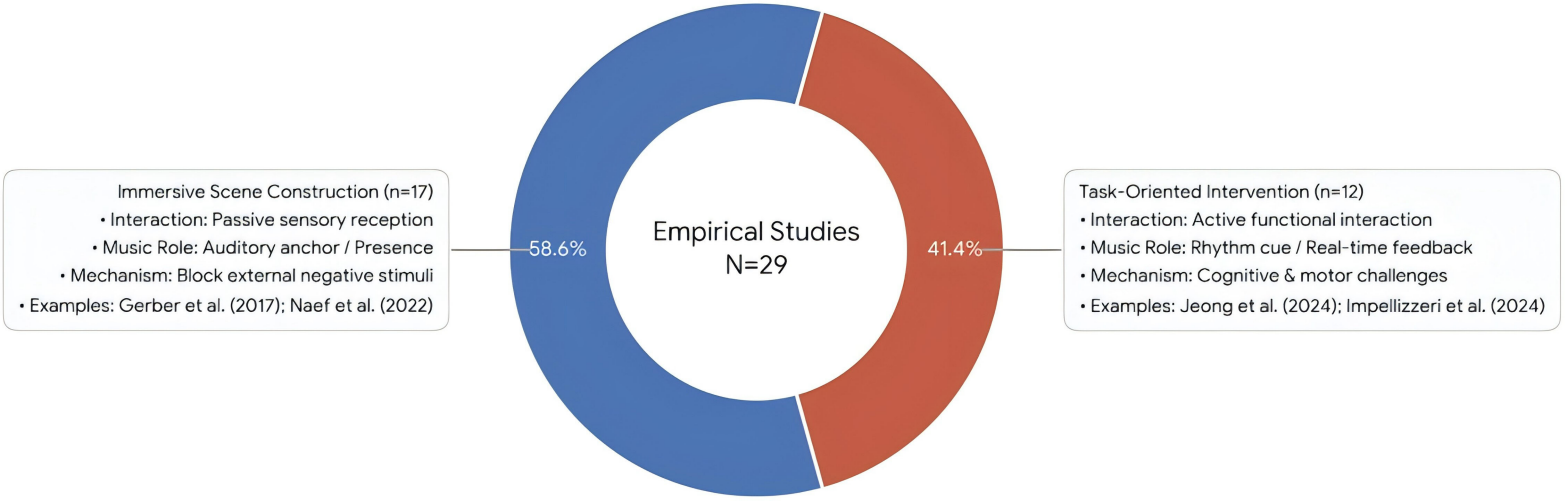
Categorization and proportion of the two primary VR-MT integration paradigms.

Within the 34 included studies, the combined application of virtual reality (VR) and music therapy (MT) exhibited significant terminological and operational heterogeneity (e.g., “music-enhanced VR therapy,” “VR with relaxing music,” or “VR-based rhythmic training”). To mitigate the risk of data redundancy arising from double-counting, a differentiated strategy was applied to the five included reviews: primary empirical data were isolated from four systematic reviews, while one highly relevant scoping review was utilized for citation tracking and validation to ensure the comprehensiveness of the empirical evidence base. Based on the level of patient interaction (passive vs. active) and the primary function of music (environmental rendering vs. movement guidance), a thematic synthesis was conducted on all original empirical protocols *(n = 29)*. As illustrated in **Figure 4**, the integration paradigms can be clearly categorized into two core frameworks: Immersive Scene Construction *(n = 17)* and Task-Oriented Intervention *(n = 12).*

The Immersive Scene Construction paradigm *(n = 17)* is fundamentally characterized by “passive sensory reception and environmental shielding.” In these interventions, patients are not required to perform specific tasks within the virtual environment. VR technology serves primarily as a tool for visual isolation, typically utilizing head-mounted displays (HMDs) to present 360° natural landscapes or tranquil virtual spaces (12, 19). Concurrently, music acts as an auditory anchor, synchronized with visual inputs to enhance the user’s sense of presence. The design objective is to leverage high-fidelity audiovisual envelopment to attenuate negative stimuli from the external environment.

In stark contrast, the Task-Oriented Intervention paradigm *(n = 12)* is defined by “active functional interaction.” Within this framework, patients transition from passive observers to active participants within the virtual environment. Here, VR provides interactive scenarios involving specific cognitive or motor challenges, while music is utilized for its structural properties—serving as rhythmic auditory cues or real-time performance feedback. For instance, Jeong et al. (16) required patients to engage in electronic drumming synchronized to a musical beat within a virtual environment to improve attentional control. Similarly, Impellizzeri et al. (14) utilized immersive VR coupled with rhythmic stimulation to enhance executive functions in individuals with Parkinson’s disease. these paradigms typically leverage software and hardware with motion capture or biofeedback capabilities and are predominantly implemented through repetitive training protocols.

#### 3.2.4 Clinical application domains and evidence of efficacy

**Figure 5:**
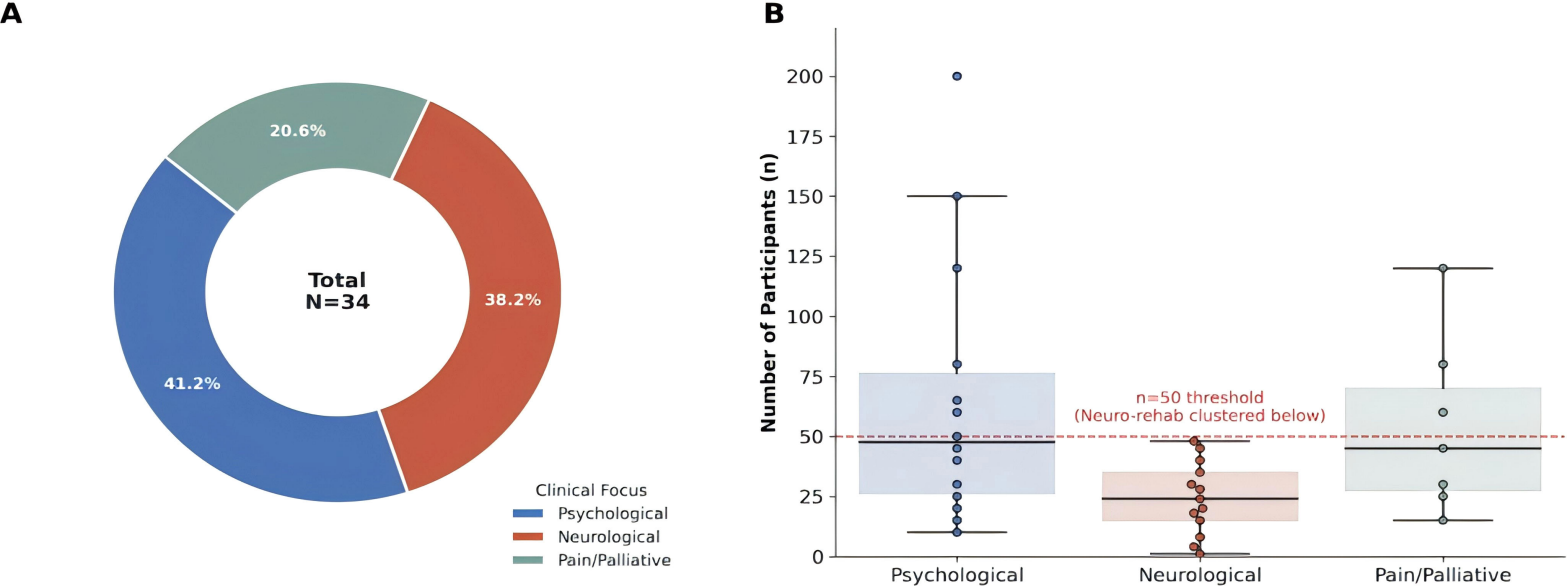
Clinical focus distribution. **(A)** Proportion of studies by clinical domain; **(B)** Distribution of sample sizes across different clinical domains.

Regarding sample sizes and participant characteristics, the included studies demonstrate substantial heterogeneity, encompassing designs ranging from single-case reports (6) to large-scale multicenter trials (37). Due to recruitment difficulties for specific clinical cohorts and the logistical costs associated with virtual reality (VR) equipment rotation, investigations in neurological and functional rehabilitation generally maintain small-to-moderate sample sizes. Clinically, the participants are distributed across three primary domains: mental health regulation (n = 14), neurological and functional rehabilitation (n = 13), and pain management and palliative care (n = 7) (**Figure 5**). These studies extensively explore both the subjective and objective clinical benefits of combined interventions. To address potential reporting bias inherent in subjective scales, multiple investigations triangulated psychological assessments with objective physiological or biological markers.

Mental health regulation represents the most widely applied clinical domain, with intervention targets highly concentrated on medical cohorts facing acute stress and healthy populations seeking psychological decompression. Studies provide evidence for the notable efficacy of combined interventions in alleviating clinical stress and improving emotional states. This literature encompasses perioperative and day-case procedures (7, 21, 37), dental settings (11, 32), high-risk pregnancy and labor (8, 40), oncology chemotherapy and palliative care (27, 28, 33, 35), as well as psychological stress management for healthy individuals and specific occupational groups (10, 13, 24, 30). Regarding objective physiological metrics, existing evidence consistently demonstrates that patients exposed to high-stress medical environments exhibit more stabilized systolic blood pressure and heart rates following immersive audiovisual interventions (7, 8, 12, 19). Furthermore, significant increases in heart rate variability (HRV), a marker of parasympathetic activation, have also been reported (36, 40).

Neurological and functional rehabilitation cohorts primarily undergo long-term interventions. This population includes patients with stroke, acquired brain injury (ABI), Alzheimer’s disease (AD), Parkinson’s disease (PD), and autism spectrum disorder (ASD). Interventions in this domain focus on cognitive and behavioral outcomes. VR-based music attention training or multisensory stimulation has been shown to improve sustained attention and unilateral neglect in patients with brain injury (16, 23, 31, 34), enhance executive functions and gait balance in patients with motor disorders (14, 26), delay memory decline in cohorts with cognitive impairment (6, 15, 18), and promote social interaction and cognitive engagement in children with ASD (9, 39). Central mechanisms revealed by neuroimaging and electroencephalography (EEG) technologies indicate active remodeling of wave rhythms in the frontal and parietal lobes during interventions, alongside explicit neurophysiological activation in the prefrontal cortex (15, 34). Notably, one study integrated the structured Neurologic Music Therapy (NMT) framework into an immersive VR environment (14). NMT differs substantially from the generic music exposure (e.g., passive listening to background music or ambient sounds) identified in most reviews; it is a clinical intervention system based on standardized neuroscience protocols and driven by specific motor or cognitive rehabilitation targets. Facilitated by VR technology, rhythmic auditory stimulation (RAS) within NMT serves as an external temporal template, forming a strong coupling with the real-time spatial visual feedback provided by VR. This mechanism effectively bypasses compromised basal ganglia networks to directly activate the motor cortex and induce cross-regional neural network reorganization, thereby elevating the intervention from mere emotional support to targeted functional training for neural pathway remodeling.

The pain management and palliative care domain focuses on vulnerable populations facing severe physical distress, encompassing patients with advanced cancer, burn rehabilitation, postoperative trauma, and those in intensive care units (ICU). Multiple studies consistently report that the multisensory distraction mechanisms of combined VR and music significantly reduce subjective pain scores in complex medical scenarios. These applications range from postoperative trauma and tooth extraction (7, 11, 32) and outpatient gynecological procedures (37) to complex pain management in advanced cancer care (17, 28, 35). Investigating the empirical evidence at a deeper micro-endocrine level, clinical trials evaluating peripheral blood samples have confirmed notable reductions in cortisol and adrenocorticotropic hormone (ACTH) levels among patients receiving VR and music interventions (38). This endocrinological outcome provides critical preliminary evidence supporting the neuroendocrine stress hypothesis that this combined therapy can modulate the hypothalamic-pituitary-adrenal (HPA) axis.

#### 3.2.5 Intervention duration and dosage

**Figure 6:**
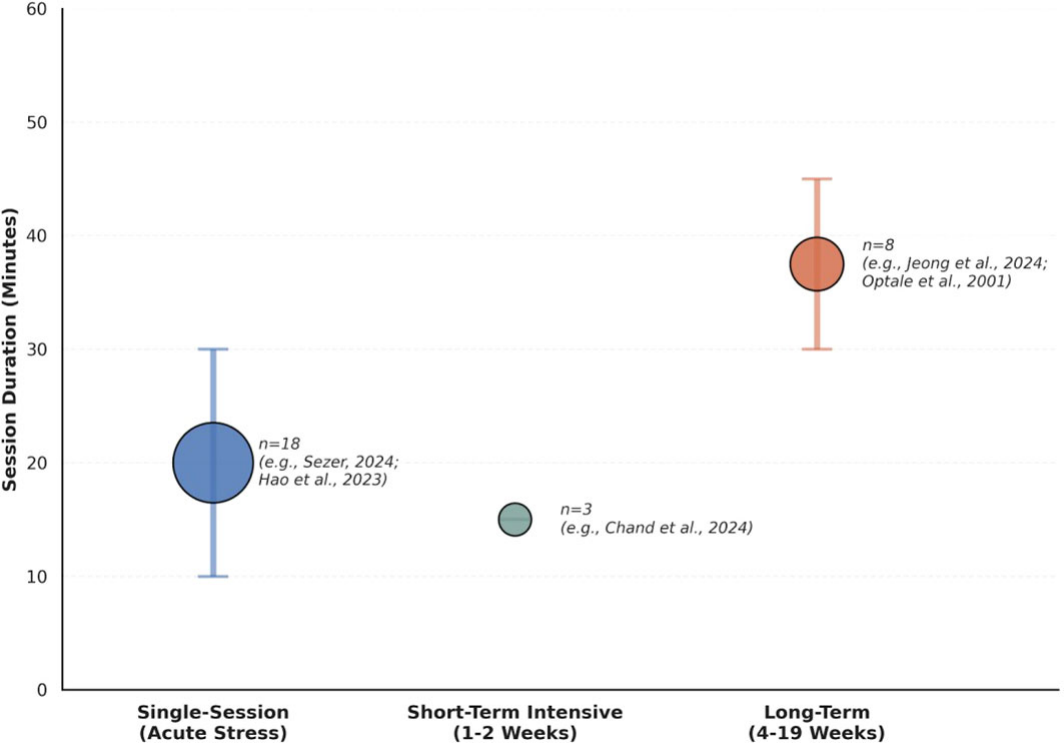
Bubble chart illustrating intervention session durations across different dosage frameworks.

The duration and dosage protocols of the interventions exhibited considerable variability, ranging from single acute sessions to longitudinal multi-session treatments with subsequent follow-up assessments (**Figure 6**). Over half of the empirical studies *(n = 18)* implemented a single-session paradigm designed to evaluate the immediate physiological and psychological effects of combined virtual reality (VR) and music interventions. Within this category, session durations were highly concentrated between 10 and 30 minutes. This acute dosage design is predominantly utilized in scenarios involving immediate clinical stressors, such as mitigating maternal anxiety during a 20-minute non-stress test (NST) (40) or serving as a rapid distraction mechanism during surgical and dental procedures (11). Extant research consistently indicates that this window of audiovisual immersion is sufficient to elicit significant parasympathetic nervous system activation (8, 19).

A minority of studies explored short-term intensive interventions spanning one to two weeks *(n = 3)*. For instance, Chand et al. (36) utilized a protocol involving 15-minute daily sessions for six consecutive days to assess stress reduction in healthy populations. These dosage frameworks are generally employed to observe cumulative treatment effects or to serve as preliminary validation for larger-scale clinical trials. Conversely, studies targeting chronic functional impairment or neurodevelopmental disorders predominantly adopted long-term intervention schemes *(n = 8)*, with durations ranging from 4 to 19 weeks. These protocols typically featured frequencies of two to three sessions per week, with each session lasting 30 to 45 minutes. Notable examples include high-frequency training (five times weekly) over eight weeks for patients with acquired brain injury (ABI) (16), a 12-week cognitive rehabilitation program (6), and a 19-week spatial-musical intervention for individuals with autism (39). Such extended dosage designs aim to facilitate neuroplastic remodeling and sustainable functional recovery through high-frequency, precision-targeted audiovisual interactive stimulation.

## 4 Discussion

Through a systematic scoping review methodology, this study synthesized 34 relevant articles published from database inception to 2024. The findings reveal that despite a growing volume of evidence in this field, systematic conceptualization of the integration paradigms between virtual reality (VR) and music therapy (MT) remains scarce. Based on a thematic synthesis of 29 studies providing primary clinical data (*empirical studies, n = 26; case reports, n = 3*), this review demonstrates that two distinct intervention paradigms, each with unique clinical adaptability, have emerged. This study aims to delineate the operational boundaries of the synergistic effects between VR and MT, thereby providing evidence-based support for individualized medical interventions. By rigorously circumventing the risk of data overlap from secondary literature and ensuring the integrity of the empirical evidence chain through citation tracking, this review comprehensively deconstructs the core data. To present a holistic overview of the field, the subsequent discussion unfolds across three progressive dimensions. First, it provides an in-depth analysis of the classification logic for combined VR and MT intervention paradigms, elucidating the synergistic architecture of visual shielding and auditory induction across different protocols. Second, it systematically elaborates on the current clinical applicability and efficacy evidence, exploring the dual-dimensional benefits—encompassing subjective perception and objective physiological responses—generated by this combined therapy within specific pathological populations. Finally, it investigates the psychophysiological mechanisms driving these clinical outcomes, thereby establishing a theoretical foundation for the design and clinical translation of future precision digital therapeutics.

### 4.1 Classification and applicable scenarios of integrated VR and MT modalities

Prior to extracting paradigms, a differentiated processing strategy was applied to the 34 articles: five reviews (four systematic reviews and one scoping review) were isolated to extract macroscopic qualitative conclusions and were utilized for citation tracking to ensure the comprehensiveness of the empirical evidence base. On this basis, a thematic synthesis was conducted on the remaining 29 studies providing primary clinical data *(empirical studies, n = 26; case reports, n = 3)*. This review demonstrates that, driven by both technological evolution and clinical demand, the integration paradigms of virtual reality (VR) and music therapy (MT) have progressively bifurcated into two complementary frameworks: Immersive Scene Construction *(n = 17)* and Task-Oriented Intervention *(n = 12)*. These paradigms have transitioned the field from rudimentary single-sensory experiences toward precision-targeted scene adaptation. Clinically, the emergence of these complementary paradigms reflects a profound response to disparities in patients’ psychophysiological energy reserves and therapeutic objectives. Immersive Scene Construction leverages the masking effects of audiovisual environments to minimize metabolic demand, primarily catering to patients in states of stress defense or pain exhaustion. Conversely, Task-Oriented Intervention requires active patient engagement, utilizing audiovisual feedback loops to trigger neuroplasticity, thereby precisely suiting rehabilitation cohorts seeking motor and cognitive functional reconstruction.

#### 4.1.1 Immersive scene construction paradigm

This paradigm constructs a synchronized “sensory sanctuary” through the profound fusion of visual environments and auditory envelopment. Its fundamental logic relies on sensory isolation to modulate the patient’s psychophysiological state (27, 38). Within the 29 primary datasets, the immersive paradigm dominated the domains of mental health (n = 10) and pain management *(n = 6)*. This high degree of mapping consistency elucidates its clinical adaptation logic: for patients undergoing perioperative procedures (11), experiencing childbirth anxiety (40), or managing acute procedural pain (32), cognitive resources are often severely occupied by stress responses. In these contexts, low-cognitive-load “passive immersion” serves as the optimal pathway for achieving immediate stabilization.

Early interventions were often constrained by limited computational power and hardware portability, relying predominantly on low-interactivity static panoramic imagery or preset audiovisual sequences (6, 12, 13, 24). However, with the advancement of wearable sensing technology, recent research exhibits an evolutionary trend toward Adaptive Closed-loop systems. Multiple frontier studies have begun integrating multimodal biofeedback, utilizing real-time neurophysiological or autonomic indicators (e.g., EEG, Heart Rate Variability [HRV]) to dynamically modulate VR environmental attributes and musical therapeutic parameters (15, 19, 36). Such designs, which adjust audiovisual parameters based on real-time bio signals, prove that personalized precision decompression can be achieved even through passive experiences. Nevertheless, the limitations of this paradigm remain significant: since the intervention relies heavily on sensory reception, its efficacy attenuates rapidly if the content is misaligned with patient preferences or if tolerability issues, such as cybersickness, arise (29).

#### 4.1.2 Task-oriented intervention paradigm

Unlike the former, which emphasizes “state regulation,” the Task-Oriented Intervention paradigm is fundamentally goal-driven and predominantly applied within the domain of neurological and functional rehabilitation. Mapping analysis reveals that all 11 studies targeting chronic neurological injuries (e.g., acquired brain injury [ABI], stroke, Parkinson’s disease [PD]) and developmental disorders (e.g., autism spectrum disorder [ASD]) employed an active interactive framework. This paradigm translates the rhythmic properties of music into temporal cues or rewarding feedback for task execution, thereby stimulating neuroplastic remodeling (14, 16). For instance, the drumming task designed by Jeong et al. (16) and the 3D spatial musical interactions developed by Vinot et al. (39) both required patients to engage in active cognitive-motor coupling over extended periods (4 to 19 weeks). The logical advantage of this “active participation” lies in its strong functional targetability, while gamified designs significantly enhance patient compliance during otherwise monotonous rehabilitation training.

Task-Oriented Interventions have increasingly advanced toward personalization and intelligent design. Virtual interactive systems integrated with automated assessment algorithms can analyze users’ cognitive states and task performance in real time utilizing technologies such as eye-tracking or motion capture, thereby dynamically adjusting the difficulty and strategy of musical interactions (9). Advanced computer-assisted rehabilitation platforms can tailor training protocols that match audiovisual rhythms with real-time physical perturbations based on an individual’s specific functional impairments (14), while gamification designs enhance patient treatment adherence through task-based reward mechanisms (22, 25, 31). These platforms typically integrate 360° immersive visual environments, multichannel acoustic fields, and dynamic motion systems equipped with force plates. The core rationale transitions from providing isolated sensory immersion to establishing highly targeted clinical rehabilitation objectives. By capturing participants’ biomechanical parameters in real time, the system dynamically adjusts audiovisual feedback cues based on task execution. This objective-driven approach deepens multisensory integration and provides a closed-loop environment for functional remodeling in patients with acquired brain injury (ABI) or Parkinson’s disease (PD) through the precise coupling of real-time physical perturbations and musical rhythms, differentiating it from traditional passive experiences. The primary advantage of such interventions lies in promoting neuroplasticity and behavioral adaptability through active participation (14, 26). However, this paradigm maintains a high reliance on technology, and individual differences—such as music preferences and task acceptability—may influence the overall intervention outcomes (10, 28, 29).

Immersive Scene Construction and Task-Oriented Intervention represent two core dimensions of the combined clinical application of VR and MT. Rather than being mutually exclusive alternatives, they demonstrate a developmental trajectory characterized by “demand differentiation and dual-track parallelism.” As evidenced by the literature statistics in this review, these two paradigms exhibit a relatively balanced distribution. The immersive paradigm, excelling in “passive sensory regulation,” accounts for a slight majority *(n = 17)*whereas the task-oriented paradigm, centered on “active functional remodeling,” also constitutes a significant proportion *(n = 12)*. In the formulation of actual clinical pathways, the selection of an intervention paradigm must strictly align with the patient’s disease trajectory and functional baseline. Driven by the deep integration of wearable sensing technologies and artificial intelligence, the absolute boundaries between these two paradigms will increasingly blur, progressively moving toward “deep fusion.” Next-generation digital therapeutics are poised to develop a composite intervention architecture combining “foundational immersive soothing” with “surface-level adaptive tasks.” This approach will furnish a high-fidelity, low-stress immersive audiovisual environment while seamlessly and dynamically embedding cognitive or motor micro-tasks based on the patient’s real-time physiological and electroencephalographic (EEG) feedback.

### 4.2 Clinical applicability and evidence of efficacy

The therapeutic efficacy of combined virtual reality (VR) and music therapy (MT) across heterogeneous clinical settings transcends simple “attention distraction,” exhibiting distinct domain clustering and symptom targetability. By synthesizing the 34 included records, this scoping review elucidates the logic underlying the clinical applicability of this combined therapy across three primary domains.

#### 4.2.1 Mental health regulation

Mental health and acute stress management represent the clinical domains with the most concentrated evidence in this field. A synthesis of existing evidence indicates that the combined intervention functions analogously to a “digital sedative” in these contexts. Compared to traditional single-modality passive music listening, the advantage of the combined approach lies in its top-down dual blockade of acute stress responses. In trials involving perioperative procedures and high-risk pregnancy non-stress tests, the intervention not only decreased subjective anxiety scores (11, 40), but physiological evaluations also provided evidence that it can notably reduce key serum stress hormone levels (38). This mechanism suggests that immersive audiovisual stimuli can occupy substantial cognitive capacity, thereby attenuating the transmission of environmental stressors to the cerebral cortex and subsequently inhibiting the overactivation of the hypothalamic-pituitary-adrenal (HPA) axis. The transition of evidence from subjective emotional scales to objective biomarkers supports the clinical utility of this combined therapy as a viable prehabilitation strategy prior to high-stress medical procedures.

#### 4.2.2 Neurological and functional rehabilitation

In the rehabilitation of neurological and functional disorders, such as acquired brain injury (ABI), Alzheimer’s disease (AD), and Parkinson’s disease (PD), efficacy evaluations have shifted from immediate emotional improvements to enduring functional remodeling. Therapeutic advances in this domain rely heavily on the mechanism of multisensory integration within Task-Oriented Interventions. When facing severe neuronal damage, conventional rehabilitation often struggles to maintain patients’ arousal levels. In these contexts, rhythmic auditory stimulation (RAS) within neurologic music therapy functions as an external temporal template, effectively bypassing the compromised basal ganglia network to directly activate the motor cortex (14). Combined with the real-time spatial visual feedback provided by VR, this strong visuo-auditory-motor coupling induces neural network reorganization across multiple brain regions. The integration of advanced computer-assisted rehabilitation platforms further enhances this process; these systems can deliver real-time physical perturbations and audiovisual feedback based on patients’ specific biomechanical parameters, embedding them deeply into the Task-Oriented Intervention paradigm. Empirical data provide evidence that training based on such integrated models significantly improves patients’ executive functions and motor execution (16, 26). Furthermore, the highly safe and motivating enriched environment provided by these interventions demonstrates clinical utility in overcoming rehabilitation plateaus among patients with chronic conditions (39).

#### 3.2.3 Pain management and palliative care (n = 7)

In the domain of pain management and palliative care, combined interventions demonstrate clinical utility in addressing complex somatic and existential distress. Pain is not exclusively a physiological sensation but rather a dual network encompassing sensory discrimination and affective motivation. In acute pain management, the combined therapy significantly elevates patients’ subjective pain thresholds by occupying cognitive bandwidth within the frontoparietal network (32, 37). Furthermore, empirical evidence in palliative care extends beyond localized pain alleviation to encompass improvements in overall quality of life and the preservation of patient dignity (29). For terminally ill patients with limited life expectancy and severe physical frailty, immersive audiovisual experiences provide a non-pharmacological form of psychological escapism. This approach effectively mitigates complex symptom clusters characterized by intertwined pain, fatigue, and depression (28, 35), thereby supporting the dual clinical and humanistic value of this digital therapeutic approach in end-of-life care.

### 3.3 Physiological and psychological mechanisms

**Figure 7:**
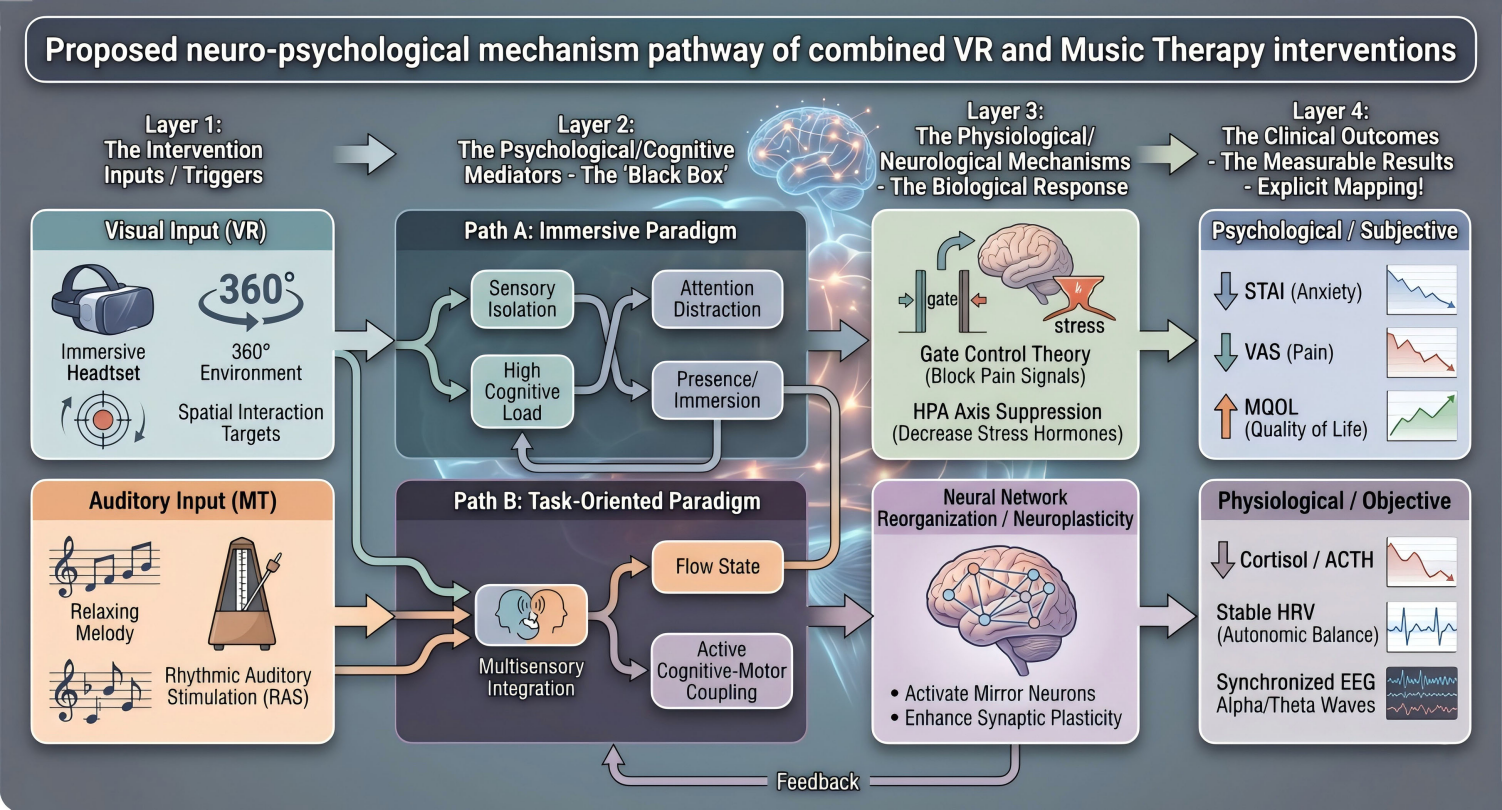
Proposed neuro-psychological mechanism pathway of combined VR and Music Therapy interventions.

Based on the synthesis of evidence from the included literature, this review constructs a synergistic mechanism model delineating the pathways from audiovisual inputs to clinical outcomes (**Figure 7**). This model identifies the visual flow and sensory isolation provided by virtual reality, alongside the auditory rhythms and emotional induction generated by music therapy, as core input sources. These elements operate within a mechanistic “black box” through two primary pathways: psychological interaction and physiological regulation. Driven by presence and immersion, the psychological pathway shifts attention allocation strategies from pathological monitoring toward the virtual experience. This shift induces an attention distraction effect and a flow state, thereby improving patient engagement and alleviating subjective anxiety and distress. The physiological pathway exhibits a dual-track framework: on one hand, for populations experiencing stress or pain, the combined intervention is hypothesized to modulate neural signal transmission via the pain gating effect. Concurrently, it engages top-down neural reflexes to inhibit the overactivation of the hypothalamic-pituitary-adrenal (HPA) axis, resulting in sedative and analgesic effects. On the other hand, for functional rehabilitation cohorts, the strong coupling of musical rhythmic stimulation and virtual spatial feedback facilitates multisensory integration. This process induces neuroplasticity and neural network reorganization, establishing a neurobiological foundation for the restoration of impaired functions.

It is important to note that the aforementioned synergistic mechanisms currently lack robust neurobiological foundations, remaining largely in a preliminary, exploratory, and hypothetical stage. To date, a limited number of studies have provided micro-level evidence using objective measures, including electroencephalography (15), functional near-infrared spectroscopy (34), and peripheral blood endocrine profiling (38). Given the substantial heterogeneity across studies regarding virtual scene interaction dimensions, musical acoustic parameters, and biomarker acquisition protocols, most mechanistic explanations rely on theoretical extrapolations and preliminary validations from small samples. Consequently, a rigorous and unified theoretical framework for audio-visual synergy has yet to be established. Therefore, future investigations require the establishment of standardized intervention and reporting guidelines, alongside the broader integration of multimodal biomonitoring technologies (e.g., simultaneous neuroimaging and continuous endocrine tracking). These methodological advancements are necessary to decode the mechanistic “black box” of combined interventions, facilitating the field’s transition from empirical adjunctive applications to precise, evidence-based digital therapeutics.

## 5 Conclusion

By systematically synthesizing evidence from *n = 34* studies, this review demonstrates the synergistic efficacy of virtual reality (VR) and music therapy (MT) in driving Immersive Scene Construction and Task-Oriented Interventions to modulate clinical stress and facilitate neurological rehabilitation. This combined paradigm provides a digital pathway for anxiety management, non-pharmacological analgesia, and interventions for cognitive impairment through mechanisms such as autonomic nervous system regulation and neuroplasticity enhancement.

However, the clinical translation of this field faces several challenges. Current empirical research generally lacks systematic evaluations of VR-related cybersickness and patient physiological tolerability. As critical variables influencing intervention adherence and safety, the relative absence of adverse effect reporting in the existing literature limits the availability of robust safety evidence for vulnerable populations. Furthermore, research is constrained by the insufficient standardization of intervention protocols, accessibility barriers imposed by hardware costs, and an incomplete understanding of how individual differences weight the synergistic mechanisms.

Future research should focus on developing stratified, standardized intervention protocols and low-cost, high-tolerability hardware. The integration of neuroimaging and multimodal biomarker monitoring will be essential to further elucidate individual variability in treatment response. Additionally, dedicated investigations into side effects such as cybersickness will provide the necessary foundation for optimizing clinical safety. By establishing multimodal interaction models through interdisciplinary collaboration, the combined application of VR and MT may transition from experimental research to widespread clinical practice, offering precise and secure digital solutions for global mental and physical health challenges.
